# Spinach consumption and nonalcoholic fatty liver disease among adults: a case–control study

**DOI:** 10.1186/s12876-021-01784-8

**Published:** 2021-05-01

**Authors:** Ebrahim Mokhtari, Hossein Farhadnejad, Ammar Salehi-Sahlabadi, Narjes Najibi, Mina Azadi, Farshad Teymoori, Parvin Mirmiran

**Affiliations:** 1 Student Research Committee, Nutrition and Endocrine Research Center, Research Institute for Endocrine Sciences, Shahid Beheshti University of Medical Sciences, Tehran, Iran; 2 Nutrition and Endocrine Research Center, Research Institute for Endocrine Sciences, Shahid Beheshti University of Medical Sciences, P.O. Box: 19395-4741, Tehran, Iran; 3 Department of Clinical Nutrition and Dietetics, Faculty of Nutrition and Food Technology, Shahid Beheshti University of Medical Sciences, Tehran, Iran; 4 Department of Nutrition, School of Public Health, Iran University of Medical Sciences, Tehran, Iran

**Keywords:** Spinach, Nonalcoholic fatty liver disease

## Abstract

**Background:**

Spinach has high antioxidants and polyphenols and showed protective effects against liver diseases in experimental studies. We aimed to assess the association between dietary intake of spinach and odds of nonalcoholic fatty liver disease (NAFLD) in a case–control study among Iranian adults.

**Methods:**

Totally 225 newly diagnosed NAFLD patients and 450 controls, aged 20–60 years, were recruited in this study. Participants’ dietary intakes were collected using a valid and reliable 168-item semi-quantitative food frequency questionnaire (FFQ). The logistic regression test was used for assessing the association between total, raw, and boiled dietary spinach with the odds of NAFLD.

**Results:**

The mean (SD) age and BMI of participants (53% male) were 38.1 (8.8) years and 26.8 (4.3) kg/m^2^, respectively. In the final adjusted model for potential confounders, the odds (95% CI) of NAFLD in individuals in the highest tertile of daily total and raw spinach intake was [0.36 (0.19–0.71), P_trend = 0.001] and [0.47 (0.24–0.89), P_trend = 0.008], respectively compared with those in the lowest tertile. Furthermore, in the adjusted analyses, an inverse association was observed between the highest yearly intake versus no raw spinach consumption and odds of NAFLD [(OR 0.41; 95% CI 0.18–0.96), P for trend = 0.013]. However, there was no significant association between higher boiled spinach intake and odds of NAFLD.

**Conclusions:**

The present study found an inverse association between total and raw spinach intake with the odds of NAFLD.

## Background

Nonalcoholic fatty liver disease (NAFLD) refers to the state of accumulation of fat in hepatocytes in persons who do not consume excessive alcohol [1]. This disease includes a wide range of conditions from fatty liver to nonalcoholic steatohepatitis (NASH), fibrosis, and cirrhosis [2]. The NAFLD pathogenesis is defined under the term “Multiple-hit theory” [1], in which several factors including genetic susceptibility, insulin resistance, adipose hormones, gut microbiome, diet, and lifestyle can affect the risk of NAFLD development. For example, it is reported some gene variants in glutathione-S-transferase [3], glutamate-cysteine ligase [4], peroxisome proliferator-activated receptors (PPARs) [5], etc. are associated with NAFLD risk in the Iranian population.

NAFLD is associated with other metabolic abnormalities, such as insulin resistance, high blood glucose level, dyslipidemia, central adiposity, and hypertension [6]. Accordingly, it is believed that NAFLD is the hepatic manifestation of metabolic syndrome. Some underlying conditions can increase NAFLD development risk, including obesity, type 2 diabetes mellitus (T2DM), and older age [7].

The average prevalence of NAFLD among the general population is estimated at 25% worldwide [8]. According to a recent meta-analysis, middle-east and Asian populations have higher NAFLD rates than the global average [9]. The prevalence in Iranian adults is reported between 20 and 50% [6] reports show a growing prevalence of NAFLD worldwide, attributed to the upward trend of adverse lifestyle changes, including unhealthy diet, sedentary behavior, and overweight [10].

Different aspects of diet, including dietary patterns, various food groups such as fruits, vegetables, and whole and refined grains, and nutrients like types of fatty acids and fructose, have been investigated with NAFLD’s risk [11, 12]. Also, the relationship between some single vegetables and chronic diseases, such as carrot and breast cancer [13], potato and diabetes [14], green leafy vegetables, and cardiovascular disease (CVD) [15], etc., have received particular attention, recently. In line with these studies, the role of spinach, as a broadleaf green, rich in nutrients, such as folates, vitamin A, C, and K, and minerals such as iron, magnesium, and manganese, and polyphenols, especially lutein, zeaxanthin, and β-carotene have been considered about the risk of NAFLD [16]. Plenty of interventional and experimental studies, have investigated spinach’s antioxidant and anti-inflammatory properties [16, 17]. To the best of our knowledge, although the association of dietary spinach with the risk of NAFLD was not assessed in observational studies, but it is shown that moderate intake of spinach may have protective effects against DNA oxidation [18]. Accordingly, it may be expected that spinach can reduce chronic disease risk, mostly related to oxidative stress. For example, some previous studies with controversial results have investigated the association of spinach with the odds of the breast (BrCa) [19, 20] and prostate cancer [21]. Furthermore, atrial stiffness [22], intrahepatic stone [23], and gallstone [24] are the other disease indicated that spinach has a protective impact against them.

A recent animal study has shown that a high spinach intake significantly reduces the adverse effects of a high-fat diet on the gut microbiome, blood glucose, lipid profile, and cholesterol accumulation in the liver [25]. Two experimental studies have also shown the anti-hyperlipidemic effects of spinach in rats fed high cholesterol diet [26] and its anti-inflammatory effects in rats with a regular diet [27].

Using common foods like spinach to improve diet quality may provide a simple and inexpensive way to reduce the risk of chronic diseases such as NAFLD. However, whether individuals receiving higher dietary spinach than those who did not have a different risk of developing NAFLD has not been assessed in previous studies. Besides, previous studies have shown that heating vegetables can have beneficial or detrimental effects on their nutritional status. Accordingly, some evidence of higher bioavailability and stability of active nutrients such as phytochemicals from cooked spinach compared to raw spinach [28, 29], suggesting that the two methods of consuming spinach may be different on the risk of liver status. Therefore, we sought to investigate the association between spinach’s dietary intake, either raw, boiled, and total, and NAFLD risk in a case–control study among Iranian adults.

## Methods

### Study population

The present study was conducted in the Metabolic Liver Disease Research Center as a referral center affiliated to Isfahan University of Medical Sciences with a case–control design. Participants were obtained through convenience sampling. Totally 225 newly diagnosed NAFLD patients and 450 controls, aged 20–60 years, were recruited in this study. NAFLD diagnosis was confirmed by the absence of alcohol consumption and other liver disease etiologies, and an ultrasonography scan of the liver was compatible with NAFLD (Grade II, III as a definite diagnosis). The control group was selected among healthy individuals based on the liver ultrasonography (not suffering from any hepatic steatosis stages).

Although Liver biopsy, recognized as the gold standard for diagnosing and staging fibrosis and inflammation, has significant limitations such as bleeding, sampling error, or interobserver variability and is not readily accepted by all patients. Nowadays, several noninvasive techniques, including biochemical and hematological tests, the scoring system using a combination of clinical and laboratory tests, Ultrasonography-based testes, and also double-contrast magnetic resonance (MR) imaging, MR elastography, and MR spectroscopy, and Diffusion-weighted MR imaging are used for diagnosis of fibrosis and hepatic inflammation [30]. Each method has its benefits and limitations. The results of laboratory and biochemical tests and scores are overlapping and cannot predict necroinflammatory activity. Despite the high accuracy of (MR) imaging techniques for quantifying the degree of necroinflammation, it is expensive and not widely available in clinical practice [30, 31]. Although the Ultrasonography test is operator-dependent and may be difficult in obese patients with narrow intercostal space, it is low cost, safe, and more accessible. A previous meta-analysis indicated that ultrasonography allows for reliable and accurate detection of the moderate-severe fatty liver compared to histology [32]. Ultrasound is likely the imaging technique of choice for screening for fatty liver in clinical and population settings, and we used an experienced physician for Ultrasonography implementation and analysis.

In the present study, patients who were referred to screen their probability of NAFLD by an Ultrasonography test because of having an abnormal or slight elevation in liver enzymes or being at risk of metabolic syndrome or having metabolic syndrome, etc. were evaluated for study eligibility criteria and patients who willing to cooperate were included in the study. The inclusion criteria for this study are not to have a special diet (due to a particular disease or weight loss) and lack of history of renal and hepatic diseases (Wilson’s disease, autoimmune liver disease, hemochromatosis, virus infection, and alcoholic fatty liver), CVD, diabetes, malignancy, thyroid disorder, and autoimmune. Also, individuals who used potentially hepatotoxic or steatogenic drugs were not included in the current study. Participants who completed less than 35 items of the food frequency questionnaire and those with under or over-reported daily energy intake (≤ 800 or ≥ 4500 kcal/d) were excluded (8 participants) and were replaced. All subjects provided written informed consent before the study enrollment.

### Dietary assessment

Participants’ dietary intakes were collected using a valid and reliable 168-item semi-quantitative food frequency questionnaire (FFQ) [33]. The FFQ listed a set of common Iranian foods with standard serving sizes. Participants were asked to express their mean dietary intake during the past year by choosing one of the following categories: never or less than once a month, 3–4 times per month, once a week, 2–4 times per week, 5–6 times per week, once daily, 2–3 times per day, 4–5 times per day, and 6 or more times a day. Portion sizes of each food item were converted into grams using standard Iranian household measures [34]. Daily energy and nutrients intake for each individual were calculated using the United States Department of Agriculture’s (USDA) Food Composition Table (FCT) [35]. The Iranian FCT was used for some traditional foods that do not exist in USDA FCT [36]. Then the consumed foods frequencies were transformed into a daily intake scale. Each serving size of dietary spinach was computed as a cup of raw spinach (30 g) and a 1/2 cup of cooked-boiled spinach (90 g) consumption, respectively (serving size calculated using USDA National Nutrient Database for Standard Reference, http://www.ars.usda.gov/ba/bhnrc/ndl).

### Anthropometric measurements

An experienced dietician performed anthropometric measurements. Weight was measured via a standard digital Seca scale (made in Germany), with minimum clothes and without shoes, and recorded to the nearest 100 g. Height was measured by a mounted non-elastic tape meter in a relaxed shoulder standing position with no shoes to the nearest 0.5 cm. Body mass index (BMI) was computed as weight (kg) divided by height in square meters (m^2^).

### Assessment of other variables

Information on other variables, including age, sex, marital status, socioeconomic status (SES), and smoking status, was collected using a standard demographic questionnaire. SES score, as an index of socioeconomic status, was calculated based on three variables, including family size (≤ 4, > 4 people), education (academic and non-academic education), and acquisition (house ownership or not). For each of these variables, participants were given a score of 1 (if their family members were ≤ 4, were academically educated, or owned a house) or given a score of 0 (if their family members were > 4, or had non-academic education, or leasehold property). The total SES score was then computed by summing up the assigned scores (minimum SES score of 0 to a maximum score of 3). Participants who had a score of 3, 2, and a sum of subjects with 1 and 0 were classified as high, moderate, and low SES, respectively.

Physical activity measurement was conducted using the International Physical Activity Questionnaire (IPAQ) through face-to-face interviews. All results of the IPAQ were expressed as Metabolic Equivalents per week (METs/week).

### Statistical analysis

Statistical analysis was performed using Statistical Package Software for Social Science, version 21 (SPSS Inc., Chicago, IL, USA). The normality of the data was tested using Kolmogorov-Smirnov’s test and histogram chart. Participants’ baseline characteristics and dietary intakes were expressed as mean ± SD or median (25–75 interquartile range) for quantitative variables and frequency (percentages) for qualitative variables. The data were compared between two groups by independent sample t test and chi-square for continuous and categorical variables, respectively. The logistic regression test was used for assessing the association between total, raw, and boiled dietary spinach with the odds of NAFLD. We first conducted a univariate analysis for each eventually confounding variable with the NAFLD for choosing the potential confounders. We entered the logistic regression analysis variables as confounders, whose p-value was lower than 0.20 [37]. Age and sex were not different between cases and controls. Based on the univariate analysis, the mentioned variables were not associated significantly with NAFLD; they do not change substantially in logistic regression results, so they were not adjusted in the analysis.

The analysis was adjusted for potential confounders, including BMI, physical activity, smoking, SES, dietary intake of energy, high-fat dairy, and other vegetables except for spinach. The odds ratio (OR) with 95% confidence interval (CI) of NAFLD across tertiles of the total, raw, and boiled dietary spinach (gram per 1000 Kcal of energy intake) was reported. P-values < 0.05 were considered statistically significant. We also conducted an additional analysis for testing the odds of NAFLD between tertiles of yearly serving size intake of raw or boiled spinach compared to the participants who do not consume raw or boiled in the last year the reference category. As only 35 participants (20 controls, 15 cases) not finished total spinach, which was too low a sample size for the reference category, we exclude the total spinach for this additional analysis.

## Results

The mean (SD) age and BMI of participants (53% male) were 38.1 (8.8) years and 26.8 (4.3) kg/m^2^, respectively. Table 1 indicates the general characteristics and dietary intake of cases and controls. Smoking was higher among NAFLD patients than in control (p value = 0.006), and also, smoking was higher among men than women (p value = 0.006, data not shown). Also, NAFLD patients had higher BMI(p value = 0.006), SES scores (p value = 0.043), and lower physical activity (p value < 0.001) than the control group. Also, the NAFLD patients had a higher dietary intake of calorie (p value = 0.006) and high-fat dairy products (p value < 0.001), whereas ate higher vegetables (p value = 0.001), total and raw spinach (p value = 0.001), and boiled spinach (p-value = 0.046) than controls. There were no significant differences between the two groups in age and sex distribution and dietary intakes of carbohydrate, protein, fat, fiber, whole grains, fruits, nuts, and legumes (P > 0.05).

**Table 1:** Characteristics and dietary intakes among cases and controls

	Cases (n = 225)	Controls (n = 450)	P value
Age (years)	38.6 ± 8.7	37.8 ± 8.9	0.293
Male, n (%)	125 (55.6)	233 (51.8)	0.354
BMI (kg/m^2^)	30.5 ± 4.0	25.0 ± 3.0	< 0.001
Smoking, n (%)	16 (7.1)	12 (2.7)	0.006
Physical activity (MET/min/week)	1119 ± 616	1590 ± 949	< 0.001
*SES, n(%)*			0.043
Low	65 (28.9)	158 (35.1)	
Middle	104 (46.2)	206 (45.8)	
High	56 (24.9)	86 (19.1)	
*Macro and micronutrients*			
Energy intake (Kcal/day)	2369 ± 621	2227 ± 645	0.006
Carbohydrate (% of energy)	55.9 ± 7.4	55.9 ± 6.5	0.917
Protein (% of energy)	13.2 ± 2.4	13.2 ± 2.2	0.941
Fat (% of energy)	30.7 ± 7.4	30.8 ± 6.5	0.927
Fiber (g/1000 Kcal)	16.7 ± 8.3	15.8 ± 6.4	0.154
Calcium (mg/1000Kcal)	528 ± 163	533 ± 153	0.723
Sodium (mg/1000Kcal)	1990 ± 1960	2040 ± 1385	0.736
Potassium (mg/1000Kcal)	1546 ± 365	1596 ± 373	0.093
*Food groups*			
Whole grains (g/day)	64.0 (30.7–115.3)	53.8 (25.1–112.8)	0.711
High fat dairy products (g/day)	237 ± 153	118 ± 107	< 0.001
Fruits (g/day)	327 ± 227	318 ± 228	0.628
Vegetables (g/day)	262 ± 138	302 ± 148	0.001
Nuts and legume (g/day)	22.1 ± 21.1	21.2 ± 18.5	0.596
Red meat (g/day)	18.1 ± 16.2	18.9 ± 17.4	0.577
Total spinach (g/day)	1.7 (0.7–3.6)	2.3 (1.0–5.6)	0.001
Raw spinach (g/day)	0.7 (0.2–1.8)	1.0 (0.5–3.0)	0.001
Boiled spinach (g/day)	0.4 (0.1–1.5)	0.6 (0.3–1.8)	0.046
Raw spinach (serving/year)	2.5 (1.2–7.6)	9.1 (3.0–22.8)	< 0.001
Boiled spinach (serving/year)	1.9 (0.6–6.3)	12.0 (6.0–36.5)	0.046

P values were computed using the independent sample t test and chi square for continues and categorical variables, respectively

Table 2 shows the association between tertiles of the total, raw, and boiled spinach consumption (g/d) and odds of NAFLD. In the crude model, all three spinach categories in the highest compared with the lowest tertile were related with lower odds of NAFLD. in the final adjusted model for confounding variables including BMI, physical activity, smoking, SES, and dietary intake of energy, high-fat dairy, and other vegetables (except spinach), the odds ratio for NAFLD in the highest compared to the lowest tertile of total spinach and raw spinach were [(OR 0.47; 95% CI 0.24–0.89), (P for trend = 0.001)] and [(OR 0.36; 95% CI 0.19–0.7), (P for trend = 0.008)], respectively. However, based on the final adjusted model, the intake of boiled spinach was no significantly associated with the odds of NAFLD [(OR 0.76; 95% CI 0.42–1.38), P for trend = 0.508)].

**Table 2:** Odds ratios (ORs) and 95% confidence intervals (CIs) for NAFLD based on tertiles of dietary spinach

	Tertiles of dietary spinach intake			P-trend
	T1	T2	T3	
*Total spinach*				
Median intake (g/day), per 1000 Kcal	0.35	1.09	3.67	
NAFLD/control	101/150	83/150	41/150	
Crude model	1.00 (Ref)	0.80 (0.55–1.16)	0.41 (0.26–0.62)	< 0.001
Model 1^†^	1.00 (Ref)	0.91 (0.55–1.52)	0.33 (0.18–0.59)	< 0.001
Model 2^‡^	1.00 (Ref)	1.13 (0.63–2.00)	0.36 (0.19–0.71)	0.001
*Raw spinach*				
Median intake (g/day), per 1000 Kcal	0.12	0.49	1.92	
NAFLD/control	97/149	83/151	45/150	
Crude model	1.00 (Ref)	0.81(0.56–1.18)	0.45 (0.29–0.68)	< 0.001
Model 1^†^	1.00 (Ref)	0.90 (0.54–1.51)	0.38 (0.22–0.68)	0.001
Model 2 ^‡^	1.00 (Ref)	1.22 (0.68–2.19)	0.47 (0.24–0.89)	0.008
*Boiled spinach*				
Median intake(g/day), per 1000 Kcal	0.06	0.30	1.14	
NAFLD/control	107/148	51/152	67/150	
Crude model	1.00 (Ref)	0.45 (0.30–0.68)	0.62 (0.42–0.91)	0.100
Model 1^†^	1.00 (Ref)	0.62 (0.36–1.07)	0.67 (0.40–1.13)	0.242
Model 2^‡^	1.00 (Ref)	0.72 (0.39–1.31)	0.76 (0.42–1.38)	0.508

^†^Model 1: adjusted for BMI, physical activity, smoking, SES, and energy intake

^‡^Model 2: Additionally adjusted for dietary intake of high fat dairy and other vegetables except of spinach

The association of yearly dietary serving of raw and boiled spinach with odds of NAFLD among tertiles of participants, who consumed these foods compared with those who had no spinach intake in the last year, was presented in Table 3. In the crude model, the odds (95% CI) of NAFLD in subjects in the highest tertile of raw and boiled spinach were 0.56 (0.33–0.95), P for trend = 0.008 and 0.60 (0.37–0.99), P for trend = 0.072 compared with those who had no consumption, respectively. In the final model, after adjusting for potential confounders, the odds (95% CI) of NAFLD in individuals in the highest tertile of raw spinach compared with those who had no consumption remained significant [(OR 0.41; 95% CI 0.18–0.96), (P for trend = 0.013)]. However, in the final model, the yearly serving intake of boiled spinach was not associated with the odds of NAFLD.

**Table 3:** Odds ratios (ORs) and 95% confidence intervals (CIs) for NAFLD according to yearly intake of raw and boiled spinach

	Categories of spinach intake				P trend
	0	T1	T2	T3	
*Raw spinach*					
Median intake (serving/year)	0	6.0	13.5	39.1	
NAFLD/control	34/57	55/104	84/139	52/150	
Crude model	1.00 (Ref)	0.89(0.52–1.53)	1.00 (0.60–1.65)	0.56 (0.33–0.95)	0.008
Model 1^†^	1.00 (Ref)	0.66 (0.32–1.38)	0.96 (0.48–1.92)	0.35 (0.17–0.73)	0.002
Model 2^‡^	1.00 (Ref)	0.76 (0.34–1.72)	1.12 (0.51–2.44)	0.41 (0.18–0.96)	0.013
*Boiled spinach*					
Median intake (serving/year)	0	1.3	3.2	12.0	
NAFLD/control	47/67	62/113	61/135	55/135	
Crude model	1.00 (Ref)	0.83 (0.51–1.35)	0.66 (0.40–1.07)	0.60 (0.37–0.99)	0.072
Model 1^†^	1.00 (Ref)	0.87 (0.44–1.72)	0.88 (0.45–1.71)	0.66 (0.34–1.30)	0.211
Model 2^‡^	1.00 (Ref)	0.72 (0.33–1.57)	0.83 (0.39–1.76)	0.69 (0.32–1.47)	0.479

^†^Model 1: adjusted for BMI, physical activity, smoking, SES, and energy intake

^‡^Model 2: Additionally adjusted for dietary intake of high fat dairy and other vegetables except of spinach

## Discussion

In the present case–control study, we found a higher total and raw spinach intake, associated with a lower odds of NAFLD. However, there was no significant association between the boiled spinach intake and the odds of NAFLD. Also, participants in the highest category of raw spinach consumption compared with those with no intake had lower odds of NAFLD; however, the highest versus no boiled spinach consumption showed any significant association with NAFLD odds.

To date, no study has examined the association of spinach intake from the usual diet with NAFLD. However, studies with conflicting results have assessed the association of spinach intake with the risk of some chronic diseases [20, 23, 24]. A previous population-based case–control study among American participants showed that individuals with a high annual intake of raw spinach had lower odds of breast cancer than those with no intake; however, the highest intake of boiled spinach versus no intake showed no association with breast cancer. Furthermore, consumption of a carrot and raw and cooked spinach twice weekly, compared to not consuming it, showed 46% lower odds of breast cancer [20]. Another case–control study among Korean women showed no relationship between higher spinach intake and breast cancer risk; however, they do not separate raw and cooked spinach analysis. The association between spinach intake and intrahepatic and gallbladder stones was investigated in two case–control studies [23, 24]. spinach consumption of two times weekly or more versus less than once monthly among Tiwanian participants was related to 65 and 84% lower odds of intrahepatic stone for men and women, respectively [23]. In contrast, a study conducted in Netherlands showed no association between spinach intake and gallstone risk [24]. A cohort study that aimed to assess the relationship between dietary antioxidant, fruit, and vegetable subclasses with the risk of prostate cancer indicated that a higher intake of vegetables rich in vitamin C includes dietary spinach along with pepper and broccoli negatively related to the risk of prostate cancer incidence [21].

Although spinach individually not assessed with NAFLD, is investigated with the risk of liver disease as a part of healthy diets such as the Mediterranean diet (MD) [38] or Dietary Approaches to Stop Hypertension (DASH) diet [39], total vegetable, or one of the main components of non-starchy vegetables, leafy green vegetables, and allium vegetables. The higher adherence to Mediterranean and DASH diets, which have a high spinach content, indicated an improvement in liver imaging, liver fibrosis score and showed an inverse association with liver diseases and related metabolic complications [38, 39]. Previously, it has been reported that vegetable consumption, especially beta-carotene-rich one, is associated with lower visceral or liver fat content and improved insulin sensitivity [40, 41]. It has been proposed that leafy green vegetables, mostly includes spinach and lettuce, have protective effects against NAFLD through prevention of intrahepatic triglyceride (IHTG) accumulation [42], hepatic steatosis, and also maintaining blood glucose, insulin, and free fatty acids in normal hematologic ranges [43]. Furthermore, some evidence supports allium vegetables’ protective effects on NAFLD and other related disorders such as hypertension, type 2 diabetes, and metabolic syndrome (MetS) [44, 45].

Despite limited evidence in human research, several animal studies have reported the possible ameliorative spinach effect on NAFLD development [25, 46]. It has been shown that spinach consumption significantly reduced the adverse effects of a high-fat diet on blood glucose, lipid profile, and cholesterol accumulation in the liver [25, 46]. Two animal studies have also observed the anti-hyperlipidemic effects in rats fed high cholesterol diet [26] and the anti-inflammatory effects of spinach, by reducing serum TNF alpha and beta levels, in rats with a regular diet [27].

Several mechanisms have been proposed to explain leafy greens’ protective effects, such as spinach on NAFLD. However, it seems that nitrate and polyphenols’ contents play a crucial role in this relationship. Supplementing the breakfast with spinach among older women resulted in an increase in the plasma values of polyphenols and carotenoids, including lutein, zeaxanthin, and β-carotene, compared with the control group also higher than strawberries and red wine [47]. Polyphenols are an important group of bioactive ingredients which a lot of advantageous effects such as hypolipidemic, anti-inflammatory, anti-fibrotic, and insulin-sensitizing properties [48]. It has also been demonstrated that polyphenols inhibit de novo lipogenesis via SREBP1c downregulation and stimulate β-oxidation in the NAFLD models [42]. Nitrate is another important bioactive compound that estimated about 80–95% of its dietary intake supplied through vegetables, mainly green leafy vegetables like spinach [49]. Previous studies have demonstrated that dietary nitrate has protective effects against inflammation and oxidative stress through its ability to activate AMPK via a rise in the xanthine oxidase-dependent NO production, and cGMP signaling declines superoxide levels through NADPH oxidases [50]. Wang et al. found that lower serum nitrate levels directly associate with aging-related liver degeneration. In contrast, dietary nitrate can restore the serum nitrate levels and reverse this process in aging mice [51]. Another animal study claimed that spinach nitrate intake could regulate lipid homeostasis, inflammatory status, and endothelial function, so it is an excellent dietary constituent for insulin resistance prevention [52].

Boiled spinach showed a non-significant association with NAFLD in the present study. Two reasons may justify it; the first is the lower consumption of boiled compared to the raw spinach (about 10% in daily intake by the gram, and about five times by serving intake per year) in the present study. Second, it is previously observed that nitrate and total polyphenolic content and antioxidant activity of some vegetables, including spinach, are reduced during the cooking process [53, 54].

Compared with other studies investigating spinach and liver disorders, our study has some advantages; we directly assessed the association of mere spinach intake independent of other vegetables with NAFLD. We also analyzed all types of spinach consumption (total, raw, and cooked). We compared NAFLD risk among participants in the highest vs. lowest intake and the highest vs. no spinach consumption.

Besides the advantages mentioned above, our study has several strengths; this study is the first study investigating the association of spinach intake as a single dietary component with the odds of NAFLD. Besides, dietary data were collected by trained interviewers in a face-to-face interview, using a validated and reproducible 168 item food frequency questionnaire (FFQ) [55], which decreases measurement bias.

We also had some limitations: firstly, the inability to discover the causal relationships and other concerns due to the study’s case–control design. Secondly, NAFLD diagnosis was based on ultrasonography, whereas the gold standard was a liver biopsy and (MR) imaging technique is more accurate. Of course, this issue can be neglected because today, due to the limitations and complications of biopsy and high cost and low availability of (MR) imaging techniques, using noninvasive methods ultrasonography is reliable and applicable in clinical practice [7]. The third limitation was no matching the cases and controls based on three essential variables, including age, sex, and BMI; however, there was no significant difference for age and sex between the cases and controls, and these variables were adjusted in analysis. We have no data on pubertal status, the number of pregnancies, hormonal conditions of participants, genetic data, etc. So, regarding the adjustment of several potential confounders, our study design cannot eliminate all potential confounding, and the effects of some residual confounding variables may have occurred.

## Conclusions

The present study found an inverse association between total and raw spinach intake with the odds of NAFLD. However, there was no significant association between higher boiled spinach intake and odds of NAFLD. Spinach is one of the richest sources of ingredients such as polyphenol and antioxidants. If its beneficial effects on chronic disease are approved in future studies, it could easily be used as a powder to enrich the nutritional values of homemade foods or products such as dairy or other foods. We suggest that our hypothesis of the association between dietary spinach and NAFLD odds be examined in more studies with higher design power, like large cohort studies and clinical trials.

## Data Availability

The data analyzed in the present study are available by the corresponding author on a reasonable request.

## Abbreviations

NAFLD: Nonalcoholic fatty liver disease
CVD: Cardiovascular disease
MetS: Metabolic syndrome
BrCa: Breast cancer
IHTG: Intrahepatic triglyceride
IR: Insulin resistance
FFQ: Food Frequency Questionnaire
SES: Socioeconomic status
IPAQ: International Physical Activity Questionnaire
USDA: United States Department of Agriculture
FCT: Food Composition Table
MD: Mediterranean diet
DASH: Dietary approaches to stop hypertension
BMI: Body mass index
SREBP1c: Sterol regulatory element-binding transcription factor 1
AMPK: AMP-activated protein kinase
cGMP: Cyclic guanosine monophosphate
NADPH: Reduced nicotinamide adenine dinucleotide phosphate
CI: Confidence interval
SD: Standard deviation
